# Quantitative evaluation of hard exudates in diabetic macular edema after short-term intravitreal triamcinolone, dexamethasone implant or bevacizumab injections

**DOI:** 10.1186/s12886-017-0578-0

**Published:** 2017-10-03

**Authors:** Yong Un Shin, Eun Hee Hong, Han Woong Lim, Min Ho Kang, Mincheol Seong, Heeyoon Cho

**Affiliations:** Department of Ophthalmology, Hanyang University College of Medicine, Seoul, South Korea

**Keywords:** Bevacizumab, Dexamethasone implant, Diabetic macular edema, Hard exudate, Triamcinolone

## Abstract

**Background:**

To quantitatively compare short-term hard exudates (HEs) alteration in patients with diabetic macular edema (DME) after intravitreal triamcinolone, dexamethasone implant or bevacizumab injections.

**Methods:**

This retrospective study enrolled DME eyes with HEs that underwent a single-dose intravitreal injection of triamcinolone (25 eyes), dexamethasone implant (20 eyes), or three monthly injections of bevacizumab (25 eyes) and completed at least three months of follow-up. All patients were examined before and after 1, 2 and 3 months of injections. Using color fundus photographs, the amount of HEs was quantified by two masked graders. The difference in HEs area between baseline and each follow-up visit was compared among the three groups.

**Results:**

After three months, HEs area was reduced to 52.9 ± 4.21% (*P* < 0.001) in the triamcinolone group, 63.6 ± 6.08% (*P* = 0.002) in the dexamethasone implant group, and 85.2 ± 5.07% (*P* = 0.198) in the bevacizumab group. A significant reduction in HEs appeared at one month in the triamcinolone group (53.5 ± 4.91%, *P* < 0.001) and at two months in the dexamethasone implant group (70.1 ± 5.21%, *P* = 0.039).

**Conclusions:**

Our study suggests intravitreal steroids (triamcinolone, dexamethasone implants) significantly reduce HEs in DME patients on short-term follow-up, whereas intravitreal bevacizumab does not. Therefore, intravitreal steroids may be useful in DME with HEs in the fovea.

## Background

Diabetic macular edema (DME) is a common cause of visual impairment in diabetic patients [1]. Macular edema can be subdivided into focal and diffuse types. The focal form results from leaking microaneurysms, which are often associated with intraretinal lipid deposition (hard exudates) in a circinate pattern [2], and the diffuse form is caused by generalized capillary hyperpermeability [3]. The proposed mechanism suggests that DME might be caused by oxidative damage, microvascular hypoperfusion, overexpression of vascular endothelial growth factor (VEGF) or inflammatory cytokines [3]. Retinal hard exudates (HEs), which can be frequently observed along with macular edema [4], are composed of lipids and lipoproteins from microaneurysms and dilated capillaries and are primarily deposited in the outer plexiform layer [5]. HEs are also known to be associated with high serum cholesterol and hemoglobin A1c (HbA1c), which can cause both photoreceptor degeneration and degeneration of neurons in the outer plexiform layer [6].

Several reports have shown that HEs could impact visual abilities. Increasing amounts of exudate appear to be independently associated with an increased risk of visual impairment [6] and the presence of HEs is considered a sign of recalcitrant macular edema with a poor prognosis for visual recovery [7]. In particular, visual outcomes are worse when HEs are deposited beneath the fovea, which may block interaction between the neurosensory retina and the retinal pigment epithelium [8]. Additionally, severe and centrally-located HEs are at increased risk of developing subretinal fibrosis, which is an infrequent complication of DME that results in further visual deterioration [9]. Accordingly, in addition to fluid component resolution, rapid resorption of HEs is also necessary in DME treatment.

There are several treatment approaches for DME with HEs. Laser photocoagulation in DME patients with HEs can reduce future visual loss but does not improve current vision [10]. While this approach reduces leakage and can eliminate HEs, exudate resorption may appear over time [11]. While intravitreal injection of triamcinolone rapidly reduces HEs and fluid [2, 12], there are associated risks of developing cataracts [13] and increased intraocular pressure [14, 15]. The impact of intravitreal injection of anti-VEGF agents on HEs remains unclear. According to Jeon and Lee [16], there were no changes in HEs after six monthly injections of bevacizumab, while Domalpally et al. [7] showed that HEs were reduced with monthly injection of ranibizumab over a two-year follow-up period. Intravitreal injection of dexamethasone implants has recently been used to treat DME, but only one published study has reported the effect on HEs [17].

Given that there have been limited studies comparing the effects of intravitreal drug injections, in particular the impact of dexamethasone implants on HEs, we decided to quantitatively compare intravitreal injection of triamcinolone acetonide, dexamethasone implant, and anti-VEGF (bevacizumab) over a short-term period using quantitative HE measurement before and after treatment. The hypothesis of this study was that there would be differences in the effectiveness of the three treatments for reducing HEs in DME patients in the short-term.

## Methods

This retrospective study included 78 eyes of 78 DME patients with HEs who visited the Retina Service at Hanyang University Guri Hospital between January 1, 2011, and June 30, 2016. The study protocol adhered to the tenets of the Declaration of Helsinki and was approved by the Hanyang University Guri Hospital Institutional Review Board/Ethics Committee.

### Patients

After reviewing electronic medical records, patients who underwent an intravitreal injection of triamcinolone (Triamcinolone acetonide; Dongkwang, Seoul, Korea; 4.0 mg/0.05 ml), dexamethasone implant (Ozurdex®; Allergan, Inc., Irvine, CA, USA; 700 μg), or three consecutive monthly injections of bevacizumab (Avastin®; Genentech, Inc., South San Francisco, CA, USA; 1.25 mg in 0.05 mL) and completed at least three months of follow-up were included in the study (triamcinolone group, dexamethasone implant group, bevacizumab group). The following criteria were used to select cases of DME with HEs: 1) no intravitreal injections or laser photocoagulation in the previous six months; 2) no evidence of neovascularization on fluorescein angiography 3) no concomitant retinal disease; 4) refractive errors lower than −6.0 or +6.0 diopters; 5) no history of retinal surgery and 6) minimal media opacity. Patients with image quality factor values lower than 60 on Topcon spectral-domain optical coherence tomography (SD-OCT) were excluded to include only fundus photographs that could be analyzed. In addition, all patients were divided into two subgroups according to the presence of HEs in the central subfield of the Early Treatment Diabetic Retinopathy Study (ETDRS) grid with a diameter of 1 mm, as the center-involving HE and non-center-involving HE groups.

### Baseline and follow-up examinations

Comprehensive ocular examinations that included log MAR best-corrected visual acuity (BCVA) testing, intraocular pressure (IOP), refractive error, slit-lamp examination, color fundus photography, SD-OCT (3D OCT-2000, Topcon, Tokyo Japan), and fluorescein angiography was performed for baseline measurement prior to injection. These procedures, with the exception of fluorescein angiography, were also conducted after 1, 2, and 3 months of injections. SD-OCT was performed using a macular cube scan (512 × 128). The standardized macular cube protocol consists of 128 horizontal B-scan lines each composed of 512 A-scans with an acquisition time of 2.4 s over a 6-mm square that was centered on the fovea. The central macular thickness (CMT), defined as the average retinal thickness in the central subfield of a standard ETDRS grid, was automatically calculated using built-in software.

Using color fundus photographs (VX-10 fundus camera, Kowa), the HEs in all patients were measured quantitatively on semi-automated imaging software using the protocol developed by Sasaki [18] described below.

### Quantitative measurement of hard exudates

**Fig. 1:**
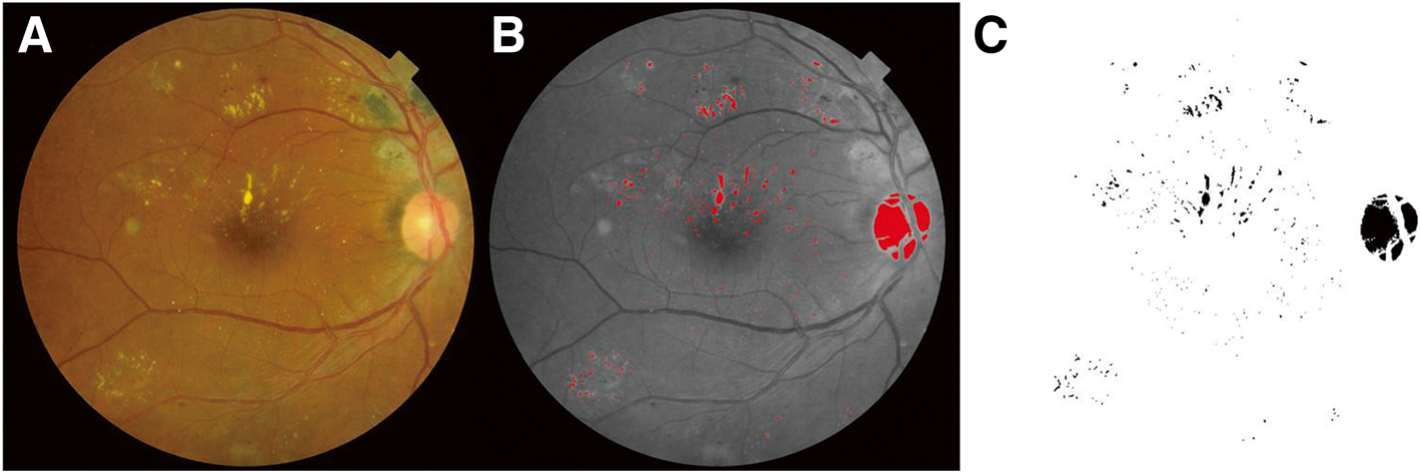
Quantitative assessment of hard exudate (HE) area using ImageJ software. Color fundus photograph **a** was split into three color channels and the total area covered by HEs was extracted from the green channel image using an automatic threshold function **b** which was converted into an automatic measurement **c** after manually eliminating areas that did not represent hard exudates (e.g., optic disc)

ImageJ software (version1.44p; available in the public domain at http://rsb.info.nih.gov/ij//; National Institutes of Health, Bethesda, MD, USA) was used in a semi-automated manner to measure the total area covered by HEs. Two masked graders (YUS, HC) individually performed this process; first, each grader measured the diameter of the optic disc (disc diameter, DD, in pixels) as a reference, given the likely differences in magnification of the retinal image due to differences in axial length, corneal curvature, and refractive error of different eyes. With the measured DD as the internal reference, we defined the ratio of the total area of HEs measured in pixels to the DD measured in pixels as the “pixel ratio.” The retinal color images were split into three color channels (red, green, and blue). The green channel was used for analysis because HEs and other retinal pathologies have better contrast in the green channel compared to the red and blue color channels [19, 20]. Total area covered by HEs was extracted as the areas identified over the threshold of intensity using an automatic threshold function (“MaxEntropy” function in ImageJ software) and areas other than HE, which were unintentionally detected, were manually eliminated by each grader independently. Finally, the total area covered by HEs was automatically calculated using the measure function (Fig. 1). If HEs were detected within the central subfield of the ETDRS grid with a diameter of 1 mm, the case was included in the “center-involving HE” group; otherwise the case was included in the “non-center-involving HE” group. As there were no significant differences between the findings of the two graders (*r* > 0.9 for all variables, intraclass coefficient), the mean of the values obtained by the two masked graders was used in analyses.

### Statistical analysis

Statistical analysis was conducted using SPSS (version 12 for Windows; SPSS Inc., Chicago, IL, USA). One-way analysis of variance (ANOVA) and chi-square test were used to compare the mean age, sex distribution, initial BCVA, IOP, spherical equivalent (SE), CMT, and HEs area among the three groups. Repeated measures ANOVA was used to compare differences in HEs area, IOP, log MAR BCVA, and CMT between baseline and each follow-up visit, and two-way repeated measures ANOVA was used to compare differences between center-involving HE and non-center-involving HE subgroups. Interobserver repeatability was examined by calculating the interclass correlation coefficient (ICC). Data are presented as mean ± standard deviation (SD). For all tests, a value of *P* < 0.05 was considered statistically significant.

## Results

A total of 70 patients that met the criteria were included in this study. Eight patients were excluded due to poor image quality of OCT and fundus photographs (4 patients), high refractive error (2 patients) and other combined retinal diseases (2 patients). Baseline characteristics of the enrolled patients are described in Table 1. The mean age of the triamcinolone group, dexamethasone implant group and bevacizumab group was 59.4 ± 10.4, 61.5 ± 10.0, and 59.1 ± 10.2 years, respectively. There were no differences in mean age, initial BCVA, IOP, SE, CMT, or HE area among the three groups. The mean baseline HEs area was 0.52 ± 0.27, 0.70 ± 0.27, and 0.55 ± 0.41 pixel ratio in the triamcinolone group, dexamethasone implant group, and bevacizumab group, respectively (*P* = 0.163, ANOVA, Table 1).

**Table 1:** Descriptive statistics for demographics and clinical characteristics of the study participants

	Triamcinolone (*n* = 25)	Dexamethasone implant (*n* = 20)	Bevacizumab (*n* = 25)	*P-*value
Gender, male%	52	50	64	0.161*
Age, years	59.4 ± 10.4	61.5 ± 10.0	59.1 ± 10.2	0.654†
BCVA, log MAR	0.64 ± 0.18	0.78 ± 0.10	0.63 ± 0.12	0.351†
IOP, mmHg	16.7 ± 2.72	16.3 ± 2.77	16.9 ± 2.41	0.478†
SE, diopter	−0.20 ± 0.77	−0.24 ± 0.66	−0.31 ± 0.58	0.844†
CMT, μm	399.2 ± 192.3	510.3 ± 200.1	451.3 ± 220.2	0.203†
HEs area, pixel ratio	0.52 ± 0.27	0.70 ± 0.27	0.55 ± 0.41	0.163†

*Chi-square; †ANOVA (One-way analysis of variance) test

*BCVA* Best corrected visual acuity, *IOP* Intraocular pressure, *SE* spherical equivalent, *CMT* Central macular thickness, *HEs* Hard exudates

Values are presented as mean ± standard deviation (SD)

**Fig. 2:**
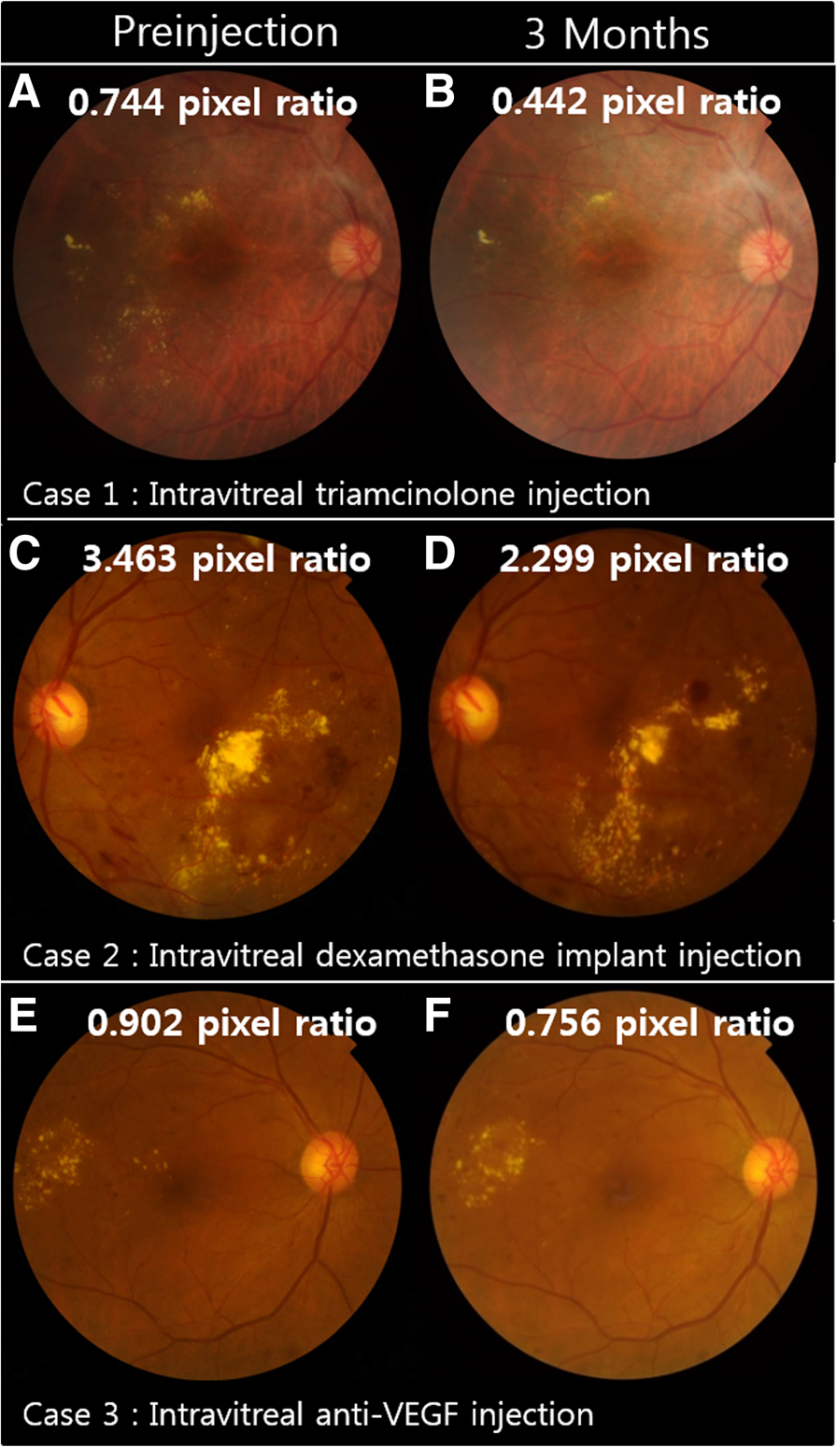
Representative cases in each group before and 3 months after intravitreal injections. The measured hard exudate area is marked in each fundus photo. **a**, **b** Intravitreal triamcinolone, **c**, **d** dexamethasone implant, and **e**, **f** intravitreal bevacizumab

**Fig. 3:**
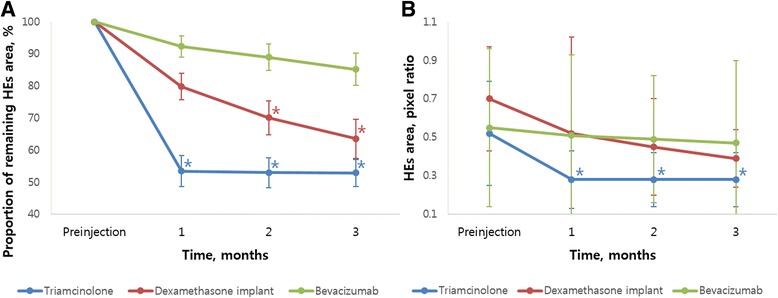
Graphs showing the change in hard exudate (HE) area in intravitreal triamcinolone, dexamethasone implant, and bevacizumab groups on monthly follow-up, presented as the proportion of remaining HE area (%, **a**) and mean HE area (pixel ratio, **b**). Asterisks indicate statistically significant differences between baseline and follow-up visits

The change in HEs area in representative cases during the follow-up period is shown in Fig. 2. In the triamcinolone group, HEs area was reduced one month after injection (proportion of remaining HEs, 53.5 ± 4.91%, *P <* 0.001, post-hoc analysis of repeated measures ANOVA) and was sustained over a three-month follow-up period. In the dexamethasone implant group, the remaining HEs area one month after injection was not statistically significant (79.8 ± 4.10%, *P* = 0.061, post-hoc analysis of repeated measures ANOVA); however, after two months of injections, the remaining HEs area was reduced to 70.1 ± 5.21% (*P <* 0.001, post-hoc analysis of repeated measures ANOVA). In the bevacizumab group, the remaining HEs area was reduced to 92.4 ± 3.31%, 89.0 ± 4.12%, and 85.2 ± 5.07% (*P* = 0.055, repeated measures ANOVA) after 1, 2, and 3 months of follow-up respectively; these differences were not statistically significant (Table 2, Fig. 3a,b). There were no statistically significant differences in the proportion of remaining HEs between center-involving HE and non-center-involving HE groups in all injection groups (Table 3).

**Table 2:** Comparison of the area of hard exudates, the proportion of remaining hard exudates, vision, and central macular thickness in each drug group (triamcinolone, dexamethasone implant, and bevacizumab group) before and after injection. The measurements at each month after injection were compared to pre-injection

		Pre-injection (1)	Month 1 (2)	Month 2 (3)	Month 3 (4)	P-value*	Post-Hoc^†^		
							1 vs 2	1 vs 3	1 vs 4
HEs area, pixel ratio	Triamcinolone	0.52 ± 0.27	0.28 ± 0.15	0.28 ± 0.14	0.28 ± 0.14	<0.001	<0.001	<0.001	<0.001
	Dexamethasone implant	0.70 ± 0.27	0.52 ± 0.50	0.45 ± 0.25	0.39 ± 0.15	0.053			
	Bevacizumab	0.55 ± 0.41	0.51 ± 0.42	0.49 ± 0.33	0.47 ± 0.43	0.150			
Proportion of remaining HEs area, %	Triamcinolone	100	53.5 ± 4.91	53.0 ± 4.66	52.9 ± 4.21	<0.001	<0.001	<0.001	<0.001
	Dexamethasone implant	100	79.8 ± 4.10	70.1 ± 5.21	63.6 ± 6.08	<0.001	0.061	<0.001	<0.001
	Bevacizumab	100	92.4 ± 3.31	89.0 ± 4.12	85.2 ± 5.07	0.055			
IOP, mmHg	Triamcinolone	16.7 ± 2.72	19.5 ± 3.19	20.7 ± 3.31	19.2 ± 2.96	<0.001	<0.001	<0.001	0.002
	Dexamethasone implant	16.3 ± 2.77	16.4 ± 4.21	16.3 ± 2.56	16.4 ± 2.71	0.984			
	Bevacizumab	16.9 ± 2.41	17.1 ± 2.18	16.5 ± 3.77	17.3 ± 2.91	0.322			
BCVA, log MAR	Triamcinolone	0.64 ± 0.18	0.42 ± 0.40	0.44 ± 0.31	0.56 ± 0.39	<0.001	<0.001	0.001	0.221
	Dexamethasone implant	0.78 ± 0.10	0.51 ± 0.35	0.53 ± 0.42	0.56 ± 0.40	0.004	0.001	0.001	0.007
	Bevacizumab	0.63 ± 0.12	0.39 ± 0.24	0.44 ± 0.27	0.40 ± 0.25	<0.001	<0.001	<0.001	<0.001
CMT, μm	Triamcinolone	399.2 ± 192.3	250.1 ± 70.3	215.2 ± 43.7	235.5 ± 50.8	<0.001	<0.001	<0.001	<0.001
	Dexamethasone implant	510.3 ± 200.1	262.8 ± 110.8	226.7 ± 101.5	218.4 ± 110.4	<0.001	0.001	<0.001	<0.001
	Bevacizumab	451.3 ± 220.2	315.9 ± 151.2	301.3 ± 153.8	312.8 ± 172.5	<0.001	<0.001	<0.001	<0.001

*Repeated measures ANOVA (analysis of variance) test; ^†^Post-hoc Bonferroni test

*HEs* Hard exudates, *BCVA* Best corrected visual acuity, *IOP* Intraocular pressure, *BCVA* Best corrected visual acuity, *CMT* Central macular thickness

Values are presented as mean ± standard deviation (SD)

**Table 3:** Comparison of center-involving HE and non-center-involving HE groups

Proportion of remaining HEs area, %		Month 1	Month 2	Month 3	
		Mean ± SD	Mean ± SD	Mean ± SD	P-value*
Triamcinolone	Total	53.5 ± 4.91	53.0 ± 4.66	52.9 ± 4.21	0.489
	Center-involving HE (*n* = 14)	54.1 ± 3.99	53.9 ± 4.03	53.3 ± 3.26	
	Non-center-involving HE (*n* = 11)	52.9 ± 4.71	52.1 ± 4.97	51.8 ± 4.85	
Dexamethasone implant	Total	79.8 ± 4.10	70.1 ± 5.21	63.6 ± 6.08	0.823
	Center-involving HE (*n* = 11)	79.7 ± 4.31	69.8 ± 5.03	61.9 ± 5.97	
	Non-center-involving HE (*n* = 9)	78.0 ± 3.17	70.3 ± 4.86	64.8 ± 6.34	
Bevacizumab	Total	92.4 ± 3.31	89.0 ± 4.12	85.2 ± 5.07	0.849
	Center-involving HE (*n* = 13)	92.3 ± 3.24	88.3 ± 3.31	84.2 ± 6.12	
	Non-center-involving HE (*n* = 12)	92.6 ± 2.53	90.5 ± 4.77	86.3 ± 4.29	

*Two-way repeated ANOVA (analysis of variance) test. *HEs* Hard exudates, Values are presented as mean ± standard deviation (SD)

**Fig. 4:**
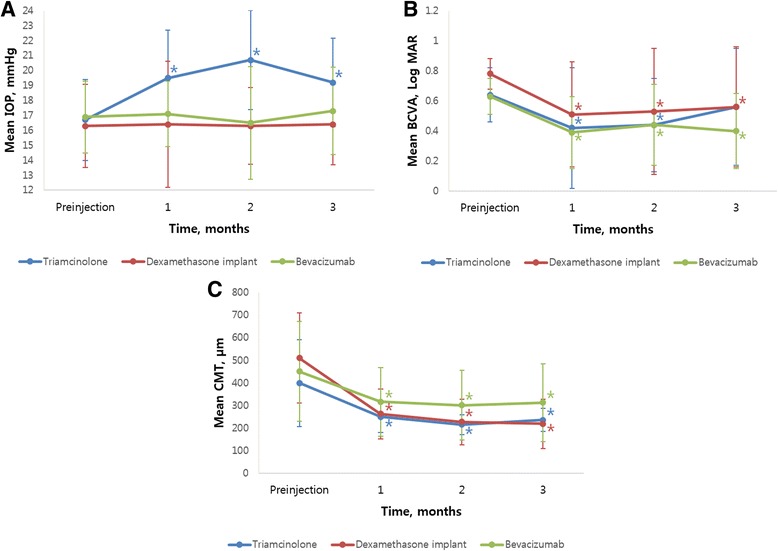
Graphs showing the change in mean intraocular pressure (IOP, **a**) best-corrected visual acuity (BCVA, **b**), and central macular thickness (CMT, **c**) in triamcinolone, dexamethasone implant, and bevacizumab groups on monthly follow-up visits. Asterisks indicate statistically significant differences between baseline and follow-up visits

IOP was increased in the triamcinolone group at the 1-, 2-, and 3-month follow-up visits, from a baseline of 16.7 ± 2.72 mmHg to 19.5 ± 3.19 mmHg, 20.7 ± 3.31 mmHg, and 19.2 ± 2.96 mmHg, respectively (*P <* 0.001, *P <* 0.001, and *P =* 0.002; post-hoc analysis of repeated measures ANOVA). In the dexamethasone implant group, there was no significant change in mean IOP, but in 4 cases IOP was elevated over 22 mmHg in the second month. In the bevacizumab group, there was no change in IOP during three months of follow-up (Table 2, Fig. 4a). There was improvement of BCVA in the triamcinolone group after one and two months of injections (*P <* 0.001 and *P =* 0.001, respectively; post-hoc analysis of repeated measures ANOVA) and in the dexamethasone implant and bevacizumab groups after 1, 2 and 3 months (dexamethasone implant group, *P* = 0.001, 0.001, and 0.007 respectively; bevacizumab group, *P* < 0.001 for all three; post-hoc analysis of repeated measures ANOVA; Table 2, Fig. 4b). CMT was reduced in all groups at every follow-up visit (all *P* < 0.05, repeated measures ANOVA; Table 2, Fig. 4c).

## Discussion

Clinically, visual improvement is the most important goal for DME. In this study, visual acuity improved in all groups in the short term; thus, reduction in HEs was investigated in addition to improvement in visual function as an additional effect. Three months after injections, intravitreal triamcinolone led to rapid and the most significant reductions in HEs over the short-term follow-up period. Intravitreal dexamethasone implant also led to a significant reduction, but this occurred more gradually than with triamcinolone; significant reduction in HEs began the first month after injection in the triamcinolone group, and the second month after implantation in the dexamethasone group. Intravitreal bevacizumab somewhat reduced the HEs area; however, this reduction was not statistically significant. There were no significant differences in center-involving HE and non-center-involving HE subgroups when all drugs were compared.

Visual improvement in the triamcinolone group persisted until two months after injection, which was in accordance with the results of a previous study [21], although other studies have shown a prolonged effect after three [12] or six months [2, 22, 23] of injections. In both the dexamethasone implant and bevacizumab groups, there was improvement in BCVA during the three months of follow-up. CMT decreased in all three groups at every follow-up. Only the triamcinolone group showed a significant increase in mean IOP over the three months of follow-up, as previous studies have reported [14, 15].

Intravitreal triamcinolone injection has been found to be an effective short-term treatment that improves visual acuity, decreases macular thickness, and reduces fluorescein leakage [13–15]. Previous studies on intravitreal triamcinolone injection have shown a rapid reduction in HEs in patients with chronic or refractory macular edema with a history of laser photocoagulation [2, 12, 22, 24]. However, there was no beneficial effect over laser photocoagulation for DME with regard to visual outcomes [25]. Furthermore, intravitreal triamcinolone injection has side effects that include increased IOP [14, 15, 26] or cataract formation [13]. The SCORE study of IOP elevation after intravitreal injection of triamcinolone for macular edema secondary to retinal vein occlusion showed IOP elevation >10 mmHg above baseline 34.0 days and 52.5 days after 1-mg and 4-mg injections of triamcinolone, respectively [26]. In this study, IOP increased significantly from the first month of intravitreal triamcinolone injection, similar to the SCORE results, although there may have been some differences in the definition of IOP elevation.

Several studies of anti-VEGF treatment on HEs have found conflicting results. Domalpally et al. [7] performed a post-hoc study of the RISE and RIDE trials and found that with up to 24 months of follow-up, more than 60% of eyes in the ranibizumab group had significant reductions in HEs starting six months after injections and an increase in VA. Similarly, a post-hoc study of the BEVORDEX trial showed that intravitreal bevacizumab injection led to a significant reduction in HEs at 12 and 24 months after injections at 4-week intervals as required [17]. On the other hand, in the study by Jeon and Lee [16], monthly intravitreal injections of bevacizumab for six months did not significantly reduce HEs.

Dexamethasone is a more potent steroid than triamcinolone [27, 28], and has been reported to be an effective drug with fewer side effects such as cataract progression or increased IOP when intravitreally implanted for DME treatment [29]. However, there have been limited studies regarding the effect of dexamethasone implant on HEs. A case report indicated that there was gradual resorption of macular exudates in Coat’s disease after six months of intravitreal dexamethasone implant injections [30]. Recently, a post-hoc study of the BEVORDEX trial reported that intravitreal dexamethasone implant injection led to a significant reduction in HEs at 12 and 24 months after injection at 16-week intervals as required [17]. However, the short-term effects could not be evaluated because fundus photography was not performed at the 3- and 6-month follow-up visits according to study protocol. In this study, we found that the intravitreal dexamethasone implant effectively reduced HEs without increasing IOP, unlike triamcinolone, after a short-term follow-up period. According to the BEVORDEX study [17], IOP increased mainly 60 days after intravitreal injection of the dexamethasone implant and almost normalized 6 months after the injection, with simple observation or the use of topical IOP-lowering eye-drops. In this study, there was no significant increase in IOP during the three months after injection. These results differ from previous research due to the small sample size of this study. In fact, a few individual cases showed IOP elevation during the second month after injection of dexamethasone implant.

Several mechanisms have been proposed to explain the effects of steroid and anti-VEGF on HEs; however, the exact mechanism of action of the corticosteroid in treating DME and HEs remains unclear. The more rapid onset of steroid’s HE-reducing effect could be explained through the following mechanisms. One possible mechanism is stabilization of the blood-retinal barrier [31, 32] by increasing levels of tight-junction proteins and inhibition of prostaglandin production through the arachidonic acid pathway as an anti-inflammatory action [33]. This may result in inhibition of proinflammatory macrophages and leukocytes, prevention of extracellular matrix remodeling, and induction of differentiation of specific anti-inflammatory macrophages with a high capacity for phagocytosis upon proinflammatory stimuli, such as lipids [34, 35]. In a recent study, HEs histopathologically composed of diffuse lipids and cholesteryl ester co-localized with apolipoprotien B and macrophages in the perivascular space [36]. A pearl necklace configuration adjacent to the HEs on the OCT has been described and assumed to be composed of lipid-laden macrophages [37]. HEs are likely resolved mainly as a consequence of reduction in leakage from multifocal capillary lesions, particularly microaneurysms. Because of its vasoconstrictive effect [33], triamcinolone might also modulate retinal hemodynamics and increase capillary wall resistance, resulting in clinical disappearance of a proportion of microaneurysms [24]. A direct disintegration effect of triamcinolone on the lipid content of HEs has also been speculated [23]. With regard to the effects of anti-VEGF on HEs, it has been proposed that pharmacologic inhibition of VEGF reduces retinal microvascular hyperpermeability [16, 38]. Additionally, steroid and anti-VEGF may have angiostatic actions through down-regulation of the production and expression of VEGF, which might play a prominent role in the formation and persistence of retinal HEs. In brief, anti-VEGF agents reduce vascular permeability by inhibiting angiogenic activity on endothelial tight junctions and the angiostatic effect. Steroids also have anti-inflammatory and vasoconstrictive effects in addition to antipermeability effects, which might result in a more rapid onset of HE reduction in the steroid treatment group.

In this study, we used a quantitative and semi-automated measurement technique to analyze alterations in HEs. Previous studies have measured HEs in a qualitative or semi-quantitative manner, or categorized them by presence or absence. Quantitative measurement of HEs using computerized methods [39, 40] or imaging software [18] might be more useful and informative than qualitative measurements in a clinical setting. Using a quantitative measurement technique on semi-automated imaging software based on the protocol developed by Sasaki [18], the effects of each treatment were evaluated and compared more objectively.

There were some limitations to this study. First, this study was retrospectively designed, not randomized, did not have a control group for comparison (i.e., a sham injection group or untreated group), and the sample size was small. Second, some patients in the current study were not treatment-naïve, and may have undergone prior intravitreal injections of other agents or laser photocoagulation, although there was a six-month wash-out period. Third, while quantitatively measuring HEs area, we manually eliminated areas other than HE. The automated thresholding method used to determine HEs area may exclude an area even though it contains HEs and include non-HEs areas, which must be manually corrected. Although the interobserver reliability showed excellent results, this may have introduced bias into this study. Finally, this study evaluated subjects for just three months after injection. Although bevacizumab did not show effective reduction of HEs compared to steroids, it may take longer to produce similar effects, as shown in 12- and 24-month result of the previous study. Additionally, it was difficult to explain the relationship between improvements in visual acuity and the reductions in HEs because of the small sample size and short-term follow-up duration. Decreased HEs would not directly reflect improved vision because the macular HE area measured in this study was not confined to the subfoveal area. The duration of subfoveal HEs may also affect visual acuity, which was not considered in this study. Therefore, additional prospective and long-term studies with large samples are needed.

Extrafoveal HEs that do not influence vision are not significantly related to DME treatment. However, foveal HEs may result in poor visual outcomes and lead to irreversible functional damage. In some cases, deposition of massive subfoveal HEs can occur after macular edema resolves [41]. The resolution of macular edema could result in HE precipitation in the macula [7], which, in severe cases, can evolve into subretinal fibrosis, with irreversible vision loss [9]. Therefore, resolution of fluid components and rapid resorption of HEs are both important in the treatment of DME patients with HEs.

## Conclusions

Our study suggests that a single dose of intravitreal steroid (both triamcinolone and dexamethasone implant) was effective for reducing HEs in DME patients in the short-term, while intravitreal bevacizumab was not. However, considering the side effects of triamcinolone, intravitreal dexamethasone implants may be a good therapeutic option for patients with DME and severe HEs in the foveal area.

## Abbreviations

BCVA: Best corrected visual acuity
BRB: Blood-retinal barrier
CMT: Central macular thickness
DME: Diabetic macular edema
ETDRS: Early Treatment Diabetic Retinopathy Study
HbA1c: Hemoglobin A1c
HEs: Hard exudates
IOP: Intraocular pressure
VEGF: Vascular endothelial growth factor
